# Atezolizumab-Conjugated Poly(lactic acid)/Poly(vinyl alcohol) Nanoparticles as Pharmaceutical Part Candidates for Radiopharmaceuticals

**DOI:** 10.1021/acsomega.2c05834

**Published:** 2022-12-12

**Authors:** Meliha Ekinci, Clenilton Costa dos Santos, Luciana Magalhães Rebelo Alencar, Hasan Akbaba, Ralph Santos-Oliveira, Derya Ilem-Ozdemir

**Affiliations:** † Faculty of Pharmacy, Department of Radiopharmacy, Ege University, Bornova, 35040 Izmir, Turkiye; ‡ Department of Physics, Federal University of Maranhão, São Luis 65080-805, Maranhão, Brazil; § Laboratory of Biophysic and Nano-Systems, Federal University of Maranhão, São Luis 65080-805, Maranhão, Brazil; ∥ Faculty of Pharmacy, Department of Pharmaceutical Biotechnology, Ege University, Bornova, Izmir 35040, Turkiye; ⊥ Nuclear Engineering Institute, Laboratory of Synthesis of Novel Radiopharmaceuticals and Nanoradiopharmacy, Brazilian Nuclear Energy Commission, Rio de Janeiro 222901-901, Brazil; # Laboratory of Nanoradiopharmaceuticals and Radiopharmacy, State University of Rio de Janeiro, Rio de Janeiro 20550-013, Brazil

## Abstract

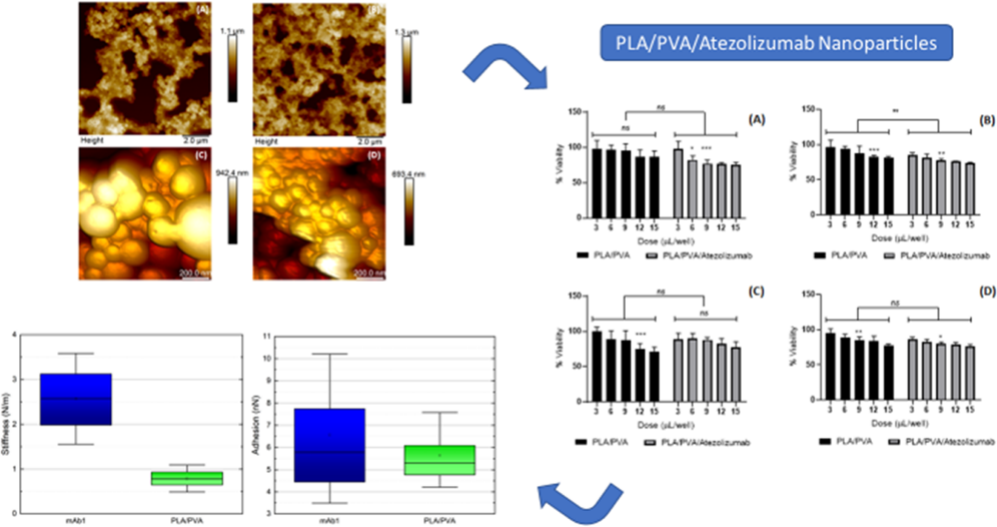

The necessity of new drugs for lung cancer therapy and imaging is increasing each day. The development of new drugs that are capable of reaching the tumor with specificity and selectivity is required. In this direction, the design of nanoparticles for tumor therapy represents an important alternative. The aim of this study was to develop, characterize, and evaluate target-specific atezolizumab-conjugated poly(lactic acid)/poly(vinyl alcohol) (PLA/PVA) nanoparticles as pharmaceutical fragment candidates for new radiopharmaceuticals. For this purpose, PLA/PVA nanoparticle formulations were prepared by the double emulsification/solvent evaporation method with a high-speed homogenizer. A special focus was oriented to the selection of a suitable method for modification of the nanoparticle surface with a monoclonal antibody. For this purpose, atezolizumab was bound to the nanoparticles during the preparation by solvent evaporation or either by adsorption or covalent binding. PLA/PVA/atezolizumab nanoparticles are characterized by dynamic light scattering, Raman spectroscopy, scanning electron microscopy, and atomic force microscopy. An *in vitro* assay was performed to evaluate the antibody binding efficiency, stability, and cytotoxicity [A549 (lung cancer cell) and L929 (healthy fibroblast cell)]. The results showed that a spherical nanoparticle with a size of 230.6 ± 1.768 nm and a ζ potential of −2.23 ± 0.55 mV was produced. Raman spectroscopy demonstrated that the monoclonal antibody was entrapped in the nanoparticle. The high antibody binding efficiency (80.58%) demonstrated the efficacy of the nanosystem. The cytotoxic assay demonstrated the safety of the nanoparticle in L929 and the effect on A549. In conclusion, PLA/PVA/atezolizumab nanoparticles can be used as drug delivery systems for lung cancer diagnosis and therapy.

## 1 Introduction

According to Adjei,^1^ lung cancer is among the top five most common cancers, with over 2.09 million cases and 1.76 million deaths worldwide. Recent studies have reported that lung cancer is more deadly than breast cancer, prostate cancer, colorectal cancer, and leukemia combined in men ≥40 years old and women ≥60 years old.^2^ Among the subtypes of lung cancer, non-small-cell lung cancer (NSCLC) makes up the majority of lung cancer cases.^3^

Drug delivery systems known as nanoparticles can be produced from substances such as polymers, proteins, lipids, ceramics, and carbon nanotubes. Polymeric nanoparticles are stable, allow for high drug molecule loading, given the chance to control the kinetics of drug release, and provide for drug targeting *via* surface-bound ligands.^4^ They are frequently used as drug and gene delivery systems due to their ability to protect drugs and against the biological environment. Also, they increase the bioavailability, safety, and biodegradability of drugs.^5−7^

In recent years, biodegradable polymers have been widely used as nanocarriers for the encapsulation of drug molecules.^8^ Poly(lactic acid) (PLA) is a polymer approved by the FDA and European regulatory authorities for biomedical applications that are widely used in the medical field for various purposes. Due to its positive properties such as biocompatibility and biodegradability, new studies continue to be conducted on the use of PLA polymer while preparing targeted drug systems.^9^ Also, poly(vinyl alcohol) (PVA) is a biodegradable semicrystalline synthetic polymer that is widely used in the pharmaceutical field to prepare solid dispersions due to its *in vitro* and *in vivo* nontoxicity studies. It is one of the most used polymers in the pharmaceutical industry due to its good compatibility with human tissues and fluids, its biodegradability, high surface stabilization, chelation properties, and low protein adsorption properties.^10^

Properties of individual polymer are key to their use for specific targets.^11,12^ It has been concluded that the use of nanocarriers for cancer treatment is promising.^13,14^ In this sense, polymeric nanoparticles could be used to deliver drugs in cancer therapy and diagnostics. In light of the above information, it was decided to use PLA and PVA polymers, which are frequently used in drug development studies in cancer diagnosis and treatment.

Atezolizumab is a monoclonal antibody approved by the FDA on October 18, 2016, for the treatment of non-small-cell lung cancer (NSCLC).^15^ Atezolizumab has an antitumoral effect by blocking the interaction of programmed death-ligand 1 (PD-L1) with programmed cell death protein 1 (PD-1) in the tumor microenvironment and preventing inhibition of T cells.^16^ It has been demonstrated that atezolizumab has fewer toxic effects than other immune checkpoint inhibitors (cytotoxic T lymphocyte-associated antigen-4 (CTLA-4) or PD-1).^17^ Although very stable,^18^ atezolizumab is an aglycosylated human IgG1. Aglycosylated antibodies are more likely to have incomplete glycosylation reactions, leading to antibody aggregation, resulting in strong ADA (anti-drug antibodies) reactions even in patients with the immune system severely damaged by chemo- or radiotherapy.^19,20^

In this study, nanosized polymeric nanoparticle formulations (PLA/PVA/atezolizumab) were prepared and characterized. Then, the cytotoxic activity of nanoparticles in A549 lung cancer cells and L929 healthy fibroblast cells were evaluated.

## 2 Materials and Methods

### 2.1 Materials

Atezolizumab (Tecentriq) was obtained from La Roche/Genentech. Poly(lactic acid) (PLA, 40–100 kDa), poly(vinyl alcohol) (PVA, 0.1% w/v, 85% hydrolyzed), and phosphate buffer (PBS) (pH: 7.4) were purchased from Sigma-Aldrich, Germany. The saline solution (0.9% sodium chloride solution, SF) was obtained from Intermountain Life Sciences, LLC. All chemicals and solvents were of either HPLC or analytical grade and were used without further purification.

### 2.2 Preparation of PLA/PVA Nanoparticles

PLA/PVA nanoparticle formulations were prepared by the double emulsification/solvent evaporation method with a high-speed homogenizer. Formulations were prepared under ideal conditions, which were determined by Ekinci et al. with quality by design (QBD) methods.^21,22^ Briefly, an aqueous solution of 1.2 mL of 1% (w/v) PVA in SF was added dropwise into 4 mL of 12.5% (w/v) PLA solution (40–100 kDa) in dichloromethane (DCM). The w/o primer emulsion was homogenized with a high-speed homogenizer for 5 min. To obtain the w/o/w double emulsion, the system was then dispersed in 10 mL of 1% PVA (w/v) solution in distilled water and homogenized under the above conditions. Afterward, the w/o/w double emulsion was gently stirred at room temperature until the solvent completely evaporated. To remove excess PVA and recover the nanoparticles, the system was redispersed in 2 mL of phosphate buffer (PBS) (pH: 7.4) and centrifuged at 20,000 rpm for 20 min at 20 °C.

To achieve PLA/PVA/atezolizumab nanoparticles to target desired cells, a special focus was oriented to the selection of a suitable method for modification of the nanoparticle surface with mAb.^23^ For this purpose, atezolizumab was bound to the nanoparticles during the preparation by solvent evaporation or by either adsorption or covalent binding.^24^

**Figure 1:**
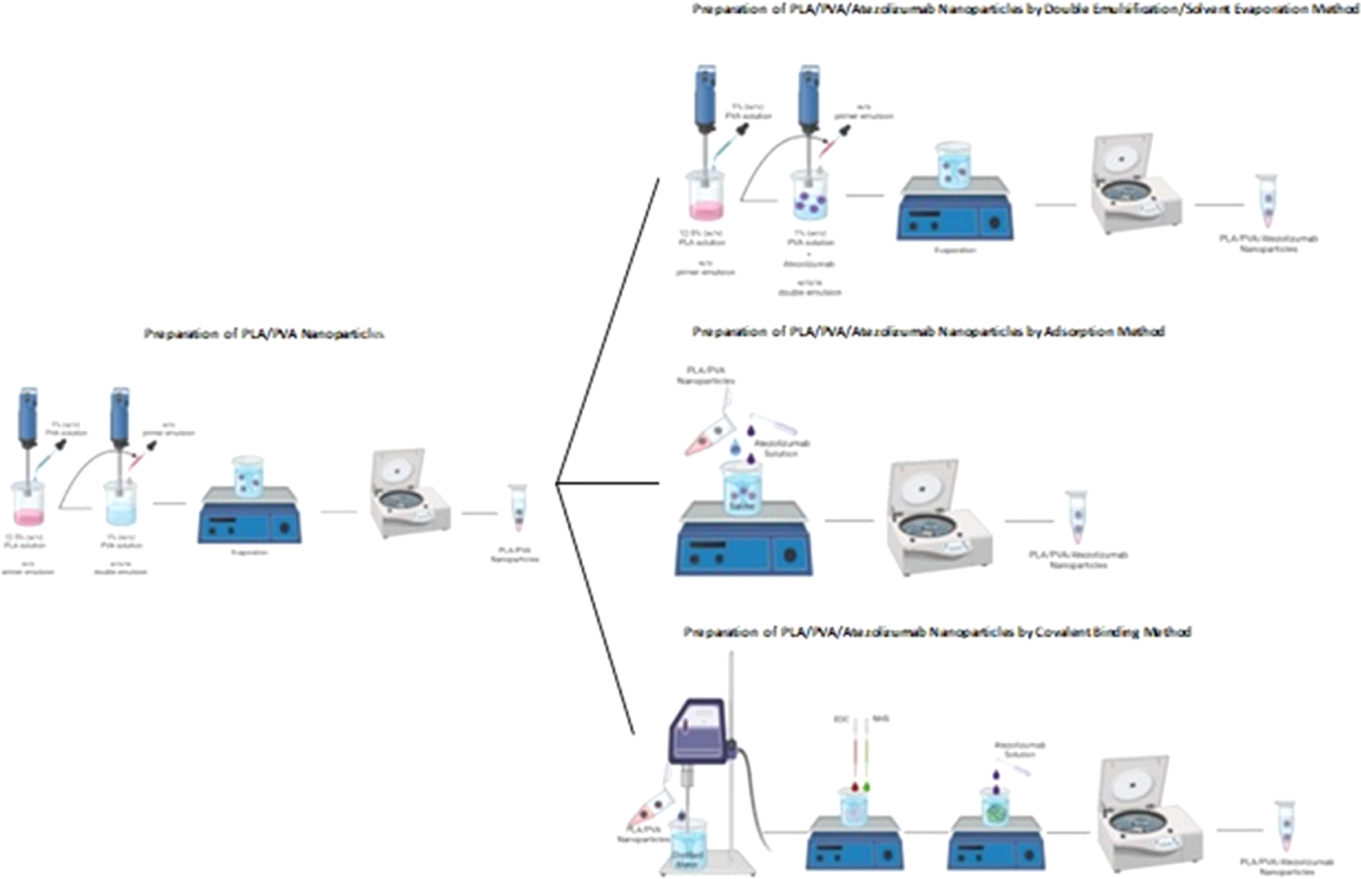
Preparation scheme of PLA/PVA and PLA/PVA/atezolizumab nanoparticles.

The preparation scheme of PLA/PVA and PLA/PVA/atezolizumab nanoparticles is shown in Figure 1.

#### 2.2.1 Preparation of PLA/PVA/Atezolizumab Nanoparticles by the Double Emulsification/Solvent Evaporation Method

The nanoparticle preparation method, which does not contain active substances, was used exactly as described above (Section 2.2). As a difference during preparation, 0.6 mL of atezolizumab was added into an aqueous solution of 0.6 mL of 0.1% (w/v) PVA.^24^

#### 2.2.2 Preparation of PLA/PVA/Atezolizumab Nanoparticles by the Adsorption Method

PLA/PVA nanoparticles were dispersed in PBS (0.6 mg·mL^–1^). Then, 600 μL of the nanoparticle suspension was mixed with 200 μL of atezolizumab solution (2 mg·mL^–1^), and the mixture was kept at 4 °C for 24 h. To remove unbound atezolizumab, the resulting dispersion of nanoparticles was centrifuged at 10,000 rpm for 15 min and washed three times with PBS.^16^

#### 2.2.3 Preparation of PLA/PVA/Atezolizumab Nanoparticles by the Covalent Binding Method

Covalent attachment of atezolizumab on the surface of the nanoparticle was accomplished by a carbodiimide functionalization reaction. First, 13.5 mg of nanoparticles was dispersed in 10 mL of distilled water at room temperature and sonicated for 5 min with continuous 155 W output power and 50/60 Hz frequency. Then, 2.5 mL of 1-ethyl-3-(3-dimethylaminopropyl) carbodiimide (EDC) (5 mg·mL^–1^) and 38.2 mg of *N*-hydroxy succinimide (NHS) (15.2 mg·mL^–1^) in distilled water were added to the nanoparticle dispersion. After stirring for 4 h at room temperature, atezolizumab (12.9 mg·mL^–1^) was added to the mixture and stirred for 18 h. To remove the unbound atezolizumab and reagents, the dispersion was centrifuged at 13,000 rpm for 5 min. The resulting particles were redispersed in 2 mL of PBS (pH: 7.4).^24^

### 2.3 Characterization Studies of Nanoparticles

#### 2.3.1 Particle Size, Distribution, and ζ Potential Analysis

Nanoparticles’ mean diameter and the width of the particle distribution (polydispersity index) were determined by a Malvern Zetasizer at room temperature and at an angle of 173°. The particle charge was quantified as the ζ potential by the Malvern Zetasizer at 25 °C, 78.5 dielectric constant, 5 mS·cm^–1^ conductivity, using a DTS 1060C Zeta cuvette, at a field strength of 40 V·cm^–1^. All measurements were made in triplicate.

#### 2.3.2 Surface and Morphological Feature Analysis

##### 2.3.2.1 Scanning Electron Microscopy

The surface morphology and size of the formulated nanoparticles were visualized by scanning electron microscopy (SEM). Before observation, the nanoparticles were coated with a gold–palladium coating on an aluminum grid; the scanning process of the coated nanoparticles was carried out at a ×12,000 magnification range and 4 kV increasing voltage conditions.^25^

##### 2.3.2.2 Atomic Force Microscopy and Nanomechanical Characterization

Solutions containing empty polymeric PLA/PVA nanoparticles and nanoparticles loaded with the atezolizumab antibody (mAb1) were investigated by atomic force microscopy (AFM) to evaluate the ultrastructure and mechanical properties. Approximately, 10 μL of solutions was deposited in previously cleaved mica and kept undisturbed until a nanoparticle film was formed. The samples were then taken to an AFM Multimode 8 (Bruker) and analyzed in PeakForce Quantitative Nanomechanics mode, using qp-HBC probes (Nanosensors) with a nominal spring constant of 0.4 N·m^–1^; however, the actual spring constants of each probe used in this work was calculated using the thermal noise method. The scans were performed with a frequency of 0.5 Hz, and a resolution of 256 × 256 pixels was used. Topography, stiffness, and surface adhesion maps were obtained for each of the analyzed samples and compared.

##### 2.3.2.3 Raman Spectroscopy

For Raman spectroscopy investigation, 30 μL of solutions containing empty PLA/PVA nanoparticles and functionalized with the atezolizumab antibody (mAb1) was deposited over a silicon substrate. The Raman spectra of the respective blanks (PVA/PLA) and functionalized nanoparticles (mAb1) were obtained using a Raman Horiba Jobin Yvon spectrometer (model iHR550). For excitation, a gas He–Ne laser (Innova 70, Coherent) with a 633.2 nm wavelength line was used and data were collected using optical fibers, detected by a Synapse CCD thermoelectrically cooled at −60 °C. An operating laser power of 2 mW was employed. Each spectrum was measured from five acquisitions of 60 s each, in each spectral region.

#### 2.3.3 Preparation Yield and Encapsulation Efficiency of PLA/PVA/Atezolizumab Nanoparticles

The preparation yield of the nanoparticles was calculated gravimetrically as a percentage using eq 1.
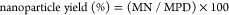
1where MN is the mass of produced nanoparticles and MPD is the mass of initial polymer materials + drug.

The amount of atezolizumab functionalized into the prepared PLA/PVA/atezolizumab nanoparticles was calculated using eq 2, based on the ratio of the amount of atezolizumab (PM) present in the nanoparticle to the amount of atezolizumab (TM) added in theory.
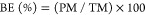
2where BE is the attachment activity, PM is the amount of drug in the nanoparticle, and TM is the amount of drug added in theory.

### 2.4 Long-Term Stability Studies of Nanoparticles

The stability of the developed formulations was determined in accordance with the stability guide at 5 ± 3 °C (refrigerator) and 25 ± 5 °C temperature, 60 ± 5% relative humidity and 40 ± 5 °C temperature, 75 ± 5% relative humidity.

In the stability study, the samples were checked for 12 months in terms of physical appearance, particle size and distribution, and ζ potentials at the beginning, at the 1st, 3rd, 6th, and 12th months.

### 2.5 Cell Culture Studies

To design a PD-L1-specific NSCLC cell-specific drug delivery system, lung cancer cell line CCL185 (A549 cells) and healthy mouse fibroblast cell line CCL1 (L929 cells) from the American Type Tissue Culture Collection (ATCC) were used. Comparative binding studies with A549 and L929 cells were performed to evaluate the cytotoxicity studies.

Cells were washed with medium and transferred to a cell culture flask. As a medium, a DMEM/F12 combination containing 50 mL of fetal bovine serum, 2 mL of l-glutamine, and 2 mL of penicillin was used. The cell culture flask was incubated at 37 ± 0.5 °C in an incubator containing 95% air-5% CO_2_ for cell growth, and the medium was changed every 48 h. When changing the medium, it was removed with disposable pipettes. The culture flask was washed with pH 7.4 PBS to get all of the parts containing the cells, and the lid was closed. Then, it was kept in the incubator for 10 min, and the PBS was removed from the culture flask. Trypsin–ethylene diamine tetraacetic acid (trypsin-EDTA) solution was put into this culture flask and kept in the incubator for 10 min. In the same way, the trypsin-EDTA solution was removed from the medium and the medium was added, and after 24 h, the medium was changed according to the above procedure. Then, the cell culture medium was renewed every 48 h. Cultivation of A549 and L929 cells was performed with a cell culture suspension medium under a laminar airflow cabinet.

PBS, trypsin-EDTA solution, and cell culture medium stored at +4 °C were kept in a water bath until it reached 37 °C. Cell culture suspensions were carefully removed from the flask opened under the laminar airflow cabinet with a disposable pipette without touching the bottom of the flask. The inside of the flask was washed two times with PBS, and the trypsin-EDTA solution was added and left in the incubator for 10 min to ensure that the cells attached to the flask surface were separated from the flask layer. The calculated amount of the cell culture medium was added to each cell culture flask, and all of this cell suspension was taken and transferred to the centrifuge tube. It was centrifuged at 3000 rpm for 3 min, and the upper collected liquid was discarded under the work cabinet and resuspended with 10 mL of medium. Cells were taken from the obtained cell suspension with small glass pipettes, and cell count was determined under a light microscope with a hemocytometer. After the cell count was calculated, the cell suspension was seeded on 96-well culture dishes for use in cytotoxicity studies.

#### 2.5.1 Cell Viability Studies

The dye exclusion test was employed to assess the viability of the cells prior to seeding. The cell suspensions were added to 25 μL of a 0.4% trypan blue solution in water, and the mixture was gently stirred. A drop of the blue mixtures was placed in a hemocytometer before being magnified 100 times under a phase-contrast microscope. The number of blue-stained cells and non-blue-stained cells were counted visually in the counting chamber for clumps and microaggregates of cells.

#### 2.5.2 Cytotoxicity–In Vitro Antitumoral Efficacy Study

The cytotoxicity studies of the formulations were evaluated by the Alamar Blue method. For this method, 100 μL of A549 and L929 cells at a concentration of 1 × 10^4^ cells·mL^–1^ were seeded into 96-well cell culture dishes separately. These cells were incubated for 24 h at 37 °C, 5% CO_2_, and in a humid environment. After 24 h, the media in the 96-well cell culture dishes were removed, and cytotoxicity studies were started. First, the cells were washed with 100 μL of PBS. Then, the formulations prepared by changing the media in the wells were added to the 96-well plate in at least six replicates as 3, 6, 9, 12, and 15 μL·well^–1^. After 24 and 48 h of incubation, cells were washed once with Dulbecco’s phosphate-buffered saline (DPBS). A complete medium containing 1/10 (v/v) Alamar Blue was prepared and added to the cells at 100 μL·well^–1^. After incubation in a 5% CO_2_ incubator for 2 h, fluorescence values at 570 and 610 nm wavelengths were measured using a multiplex reader, and the % viability values were calculated by comparing them with the unexposed control group. Cell viability was calculated using eq 3
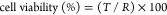
3where *T* is the absorbance value read from the tested samples and *R* is the absorbance value read from the control group.

### 2.6 Statistical Analysis

Statistical analyses and variance analyses of all *in vitro* results were performed using the GraphPad Prism 6.0 and SPSS (version 25) program. All data were given as mean and ±standard error of the mean (mean ± SD). Data were evaluated by one-way analysis of variance (ANOVA). Tukey’s multiple comparison test was applied to the groups when the *p* values were found to be less than 0.05 in the comparison between the groups. SPSS (version 25) software was used for all statistical comparisons and graphs. *p* values less than 0.05 were accepted as an indication that the difference between the groups compared was statistically significant.

## 3 Results and Discussion

### 3.1 Preparation of PLA/PVA/Atezolizumab Nanoparticles

PLA/PVA nanoparticles were successfully prepared by the solvent evaporation method. Surface-functionalized PLA/PVA/atezolizumab nanoparticle formulations were prepared by solvent evaporation (M1), adsorption (M2), and covalent binding (M3) methods. The ideal method was determined by comparing the results of the characterization studies of the formulations prepared using different preparation techniques.

### 3.2 Characterization Studies of PLA/PVA/Atezolizumab Nanoparticles

#### 3.2.1 Particle Size, Distribution, and ζ Potential Analysis

The results of particle size, distribution, and ζ potential of PLA/PVA (blank) and PLA/PVA/atezolizumab nanoparticle formulations (M1, M2, and M3) are given in Table 1.

**Table 1:** Particle Size, Distribution, ζ Potential, Preparation Yield, and Encapsulation Efficiency of Nanoparticle Formulations

formulation	particle size (nm ± ss)	PdI (±ss)	ζ potential (mV ± ss)	preparation yield (%)	encapsulation efficiency (%)
blank	181.7 ± 2.194	0.104 ± 0.049	–0.88 ± 0.45	65.38	
M1	248.7 ± 2.116	0.132 ± 0.084	–2.15 ± 0.27	62.50	75.56
M2	239.1 ± 1.626	0.148 ± 0.022	–0.87 ± 0.55	56.74	53.04
M3	289.9 ± 12.66	0.178 ± 0.077	–4.02 ± 1.56	64.85	30.94

The size of the nanoparticles has a vital role in the uptake of nanomedicine in tumor tissue.^26,27^ As expected, there was a slight increase in particle size after mAb loading. However, despite this increase, all three formulations have suitable physicochemical properties for tumor targeting. When the size, PdI, and ζ potential values of the formulations prepared with different methods (M1–3) were compared, no significant difference was found (*p* < 0.05). Also, it is important to notice that a PdI value less than 0.3 represents a monodispersive behavior of the nanoparticles and corroborates the use for pharmaceutical products.^28,29^

#### 3.2.2 Surface and Morphological Feature Analysis

##### 3.2.2.1 Scanning Electron Microscopy

**Figure 2:**
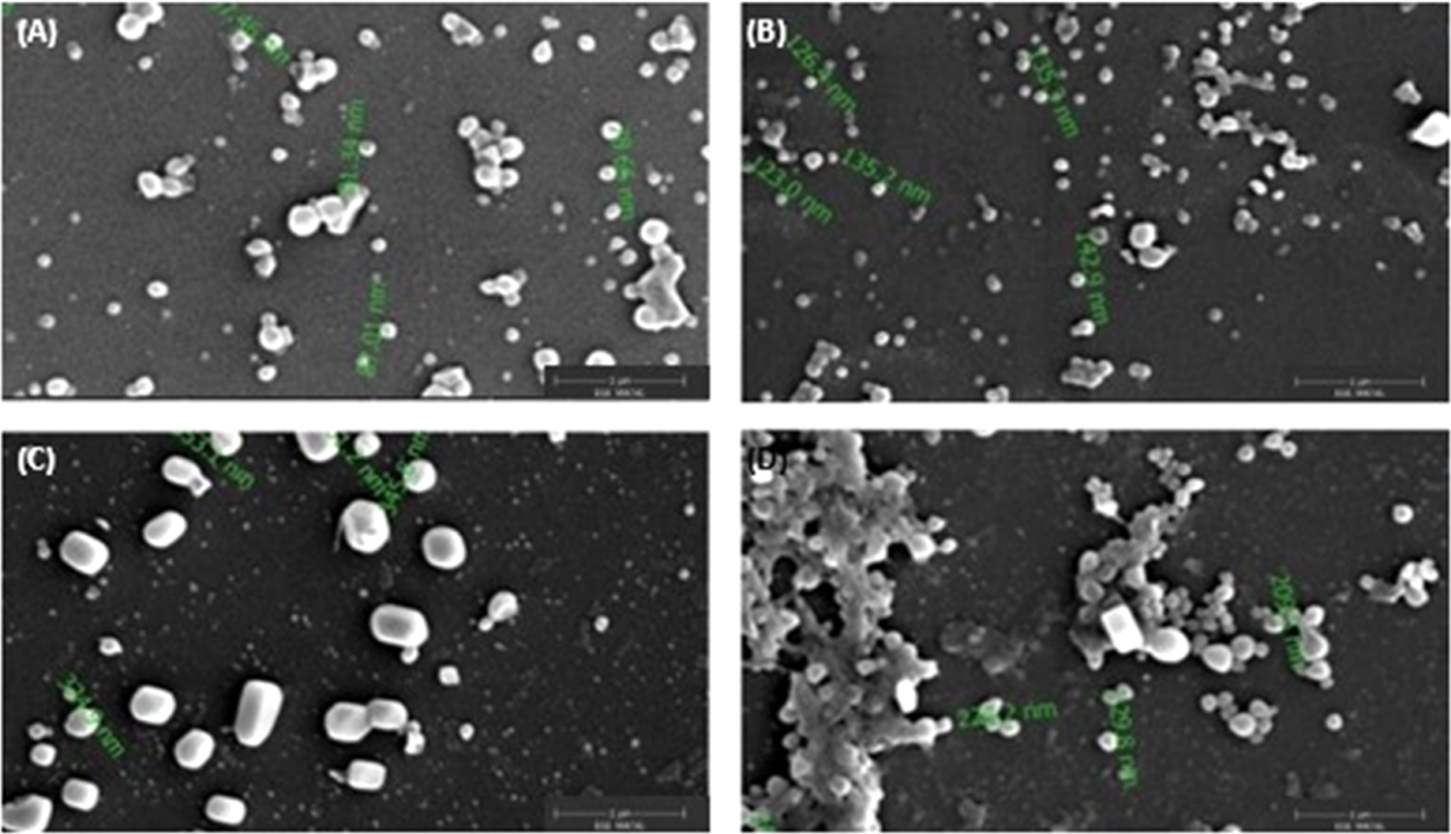
SEM images of nanoparticle formulations: (A) PLA/PVA nanoparticle formulation (blank); (B) PLA/PVA/atezolizumab nanoparticle formulations by the solvent evaporation method (M1); (C) PLA/PVA/atezolizumab nanoparticle formulations by the adsorption method (M2); and (D) PLA/PVA/atezolizumab nanoparticle formulations by the covalent binding method (M3).

The SEM images of PLA/PVA nanoparticle formulation (blank) and PLA/PVA/atezolizumab nanoparticle formulations (M1, M2, and M3) are given in Figure 2.^21,22^

As seen in Figure 2, the dimensions of the particles were compatible with the results of DLS analysis; they were spherical in shape with a smooth surface, and the particles in the formulation prepared by the covalent bonding method (M3) were in the aggregate form. The aggregation process can be explained by the high surface area-to-volume ratio of nanoparticles, resulting in reactive and colloidal instability.^30^

##### 3.2.2.2 Atomic Force Microscopy and Nanomechanical Characterization

**Figure 3:**
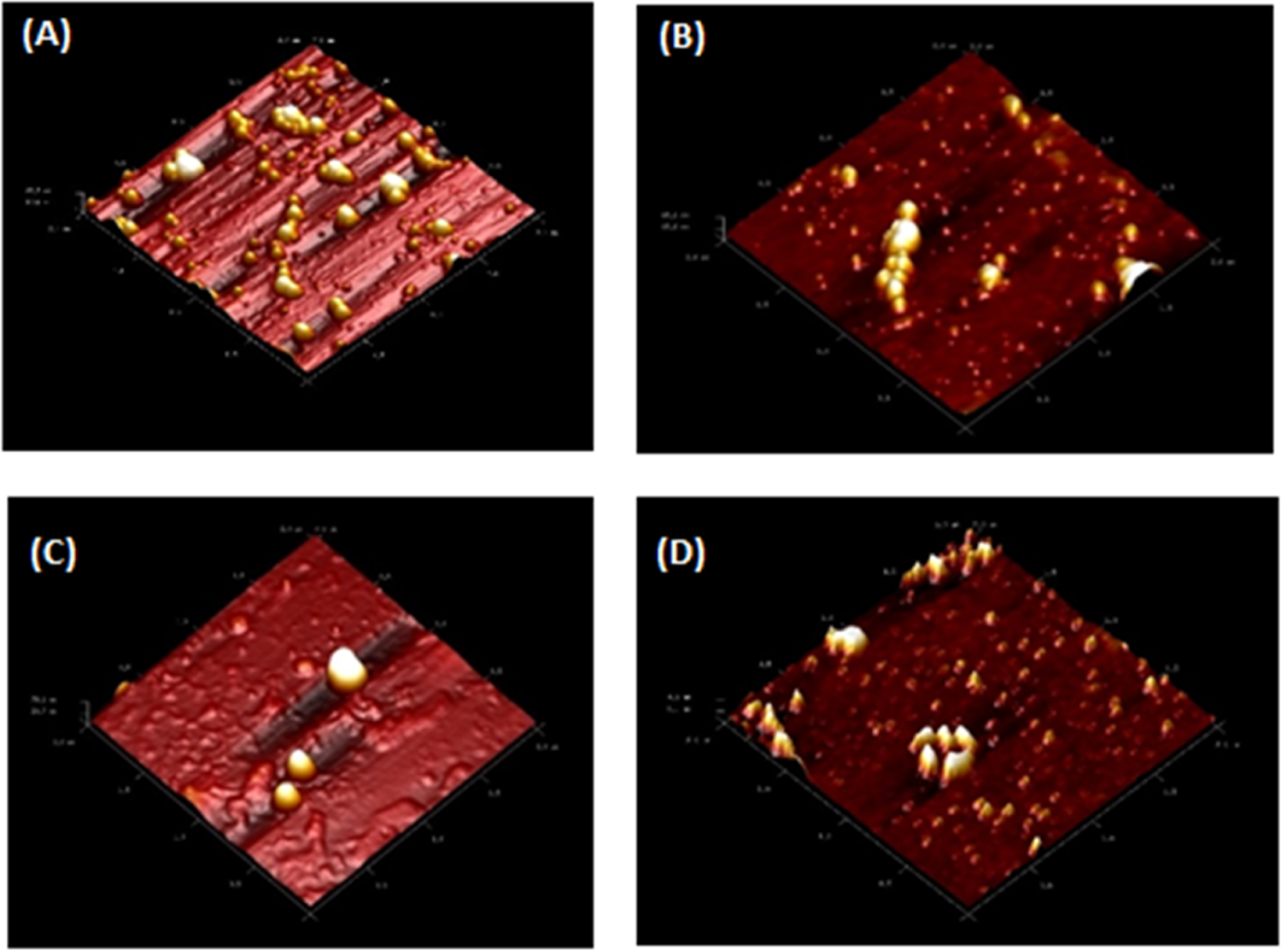
AFM images of nanoparticle formulations. (A) PLA/PVA nanoparticle formulation (blank), (B) PLA/PVA/atezolizumab nanoparticle formulations by the solvent evaporation method (M1), (C) PLA/PVA/atezolizumab nanoparticle formulations by the adsorption method (M2), and (D) PLA/PVA/atezolizumab nanoparticle formulations by the covalent binding method (M3).

The AFM images of PLA/PVA nanoparticle formulation (blank) and PLA/PVA/atezolizumab nanoparticle formulations (M1, M2, and M3) are given in Figure 3. As seen in Figure 3, the images of the particles were compatible with the SEM results, spherical in shape with a smooth surface, and the particles in the formulation prepared by the covalent bonding method were in the aggregate form.

In accordance with the results of characterization studies (Section 2.3), it was decided to use the solvent evaporation technique (M1) for further studies.

**Figure 4:**
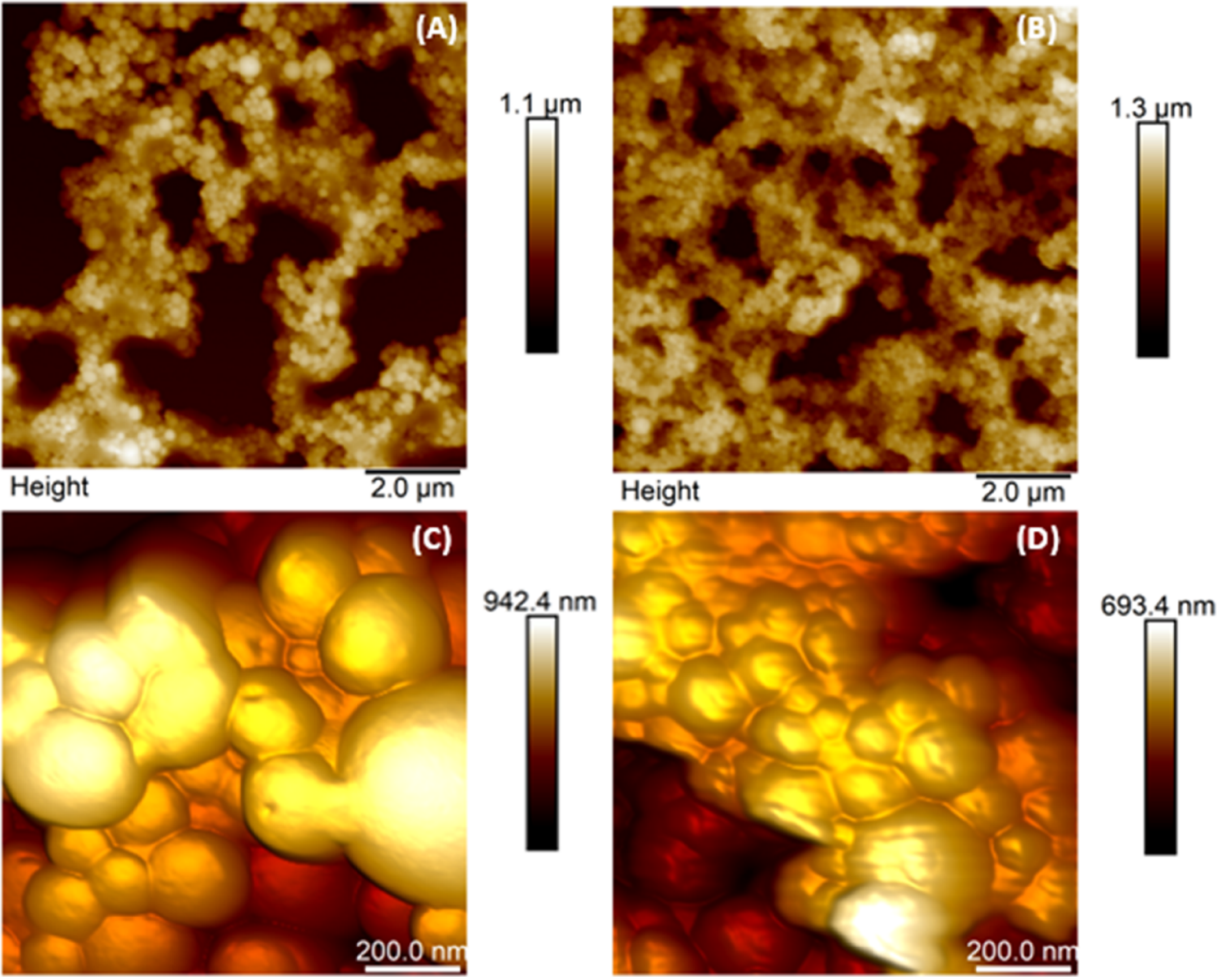
Topography images of AFM in polymeric PLA/PVA nanoparticles (A, C) and PLA/PVA/atezolizumab nanoparticles (B, D). Images (A) and (B) are 10 micron scans. Images (C) and (D) are 1 μm scans over a region containing only nanoparticles (avoiding the substrate). The scale bar shows the height differences between the lowest and highest structures in the image.

Figure 4 shows the topography maps of the PLA/PVA (Figure 4A,C) and PLA/PVA/atezolizumab nanoparticles (Figure 4B,D). Both samples present uniform size distribution, as seen in the 10 micron scan images (Figure 4A,B). The higher-resolution image of the sample without the antibody shows nanoparticles with a uniform surface (Figure 4C), while the higher-resolution image (Figure 4D) of the PLA/PVA/atezolizumab nanoparticles shows changes in the particle surface texture, which may be associated with the presence of its internal and external contents.

**Figure 5:**
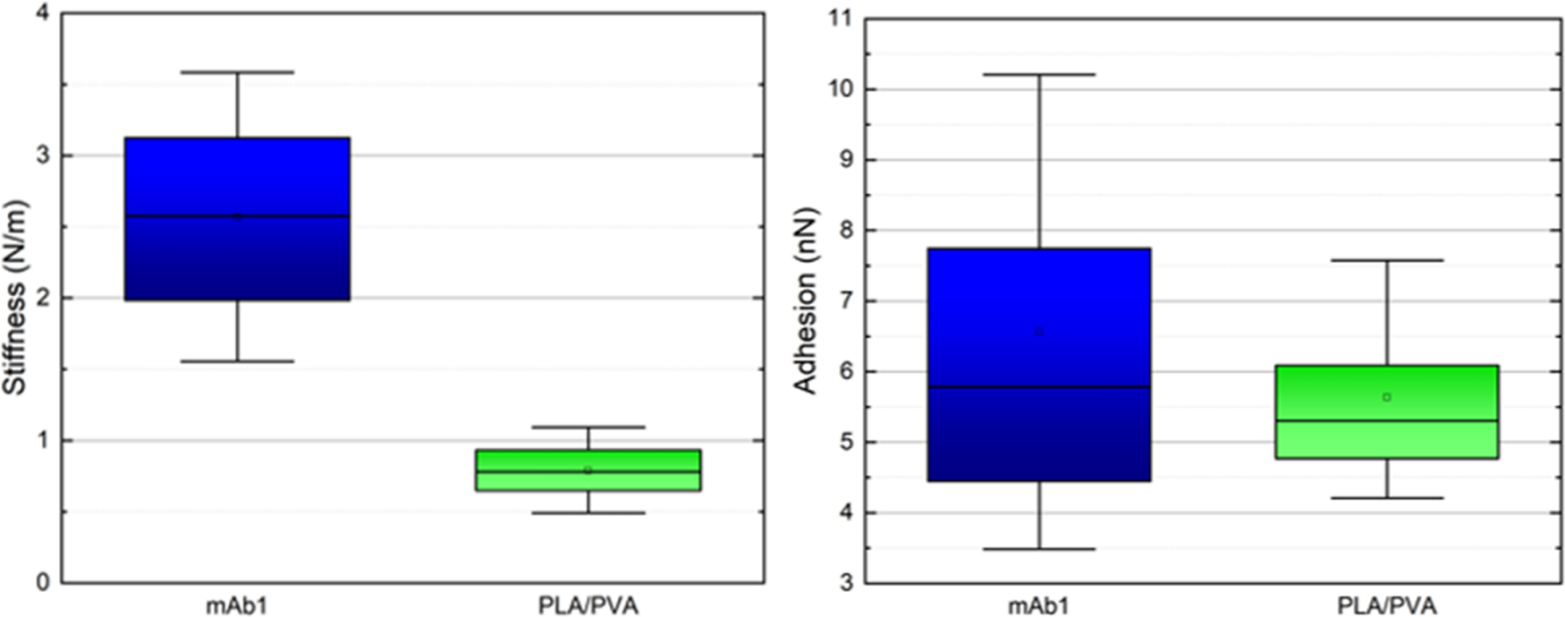
Stiffness box chart (left) of nanoparticles loaded with atezolizumab antibody (mAb1: blue) and blank (PLA/PVA: green). The average stiffness values are 0.79 and 2.57 N·m^–1^, respectively. Adhesion box chart (right) of nanoparticles, loaded (blue) and empty (green). The average adhesion force values are 6.56 and 5.63 nN, respectively.

To confirm the atezolizumab antibody loading of the nanoparticles, indentation tests were performed on both samples to compare their stiffness. In regions containing only nanoparticles (without considering the substrate in the scan), more than 65,000 force curves were acquired, and from them, the stiffness data were calculated with huge indentation statistics. The graph shown in Figure 5 (left) presents the result for each sample. After the loading process, the nanoparticles containing the atezolizumab antibody have a stiffness approximately 3 times the stiffness of the blank nanoparticles (2.57 and 0.79 N·m^–1^, respectively), confirming the effectiveness of the encapsulation process, since a filled structure is less deformable and, therefore, more rigid when compared to an empty structure. The use of AFM for encapsulation of drugs in nanoparticles^31^ as for pharmaceuticals products analysis^32^ has been demonstrated previously. For instance, Dos Reis et al.^28^ have used AFM to determine the encapsulation of dacarbazine and phthalocyanine in polymeric nanoparticles.^28^

Another interesting point to be observed in Figure 5 (left) is the dispersion of the values. For the PLA/PVA nanoparticles, the stiffness values are uniform and concentrated around the average value (0.79 N·m^–1^), while for PLA/PVA/atezolizumab nanoparticles, the size of the box is larger, showing variability in the sample stiffness (with an average value of 2.57 N·m^–1^). This may be associated with differences in the amount of encapsulated antibody in each particle.

To confirm the nanomechanical changes on the surface of the nanoparticles after the inclusion of the atezolizumab antibody, from the force curves, values of nonspecific surface adhesion were also evaluated. The adhesion force is measured through the deflection of the cantilever, and the pullout force is defined as the maximum attraction force during the retraction of the AFM tip exiting from the sample surface. In the general case, the adhesion force is a combination of the electrostatic force, van der Waals force, capillary force, and forces due to chemical bonds. In our case, unspecific interaction, the differences in the adhesion maps are composed mainly of electrostatic forces since these are of greater intensity and both samples are in the same environmental conditions; therefore, there are no significant differences in the meniscus adhesion. The graph shown in Figure 5 (right) presents the adhesion force values for each sample. It is possible to observe that the average adhesion value of the samples is changed after atezolizumab antibody surface functionalization (6.56 and 5.63 nN for loaded and unloaded nanoparticles, respectively). This result is related to the presence of antibodies on the outside of the particle.

**Figure 6:**
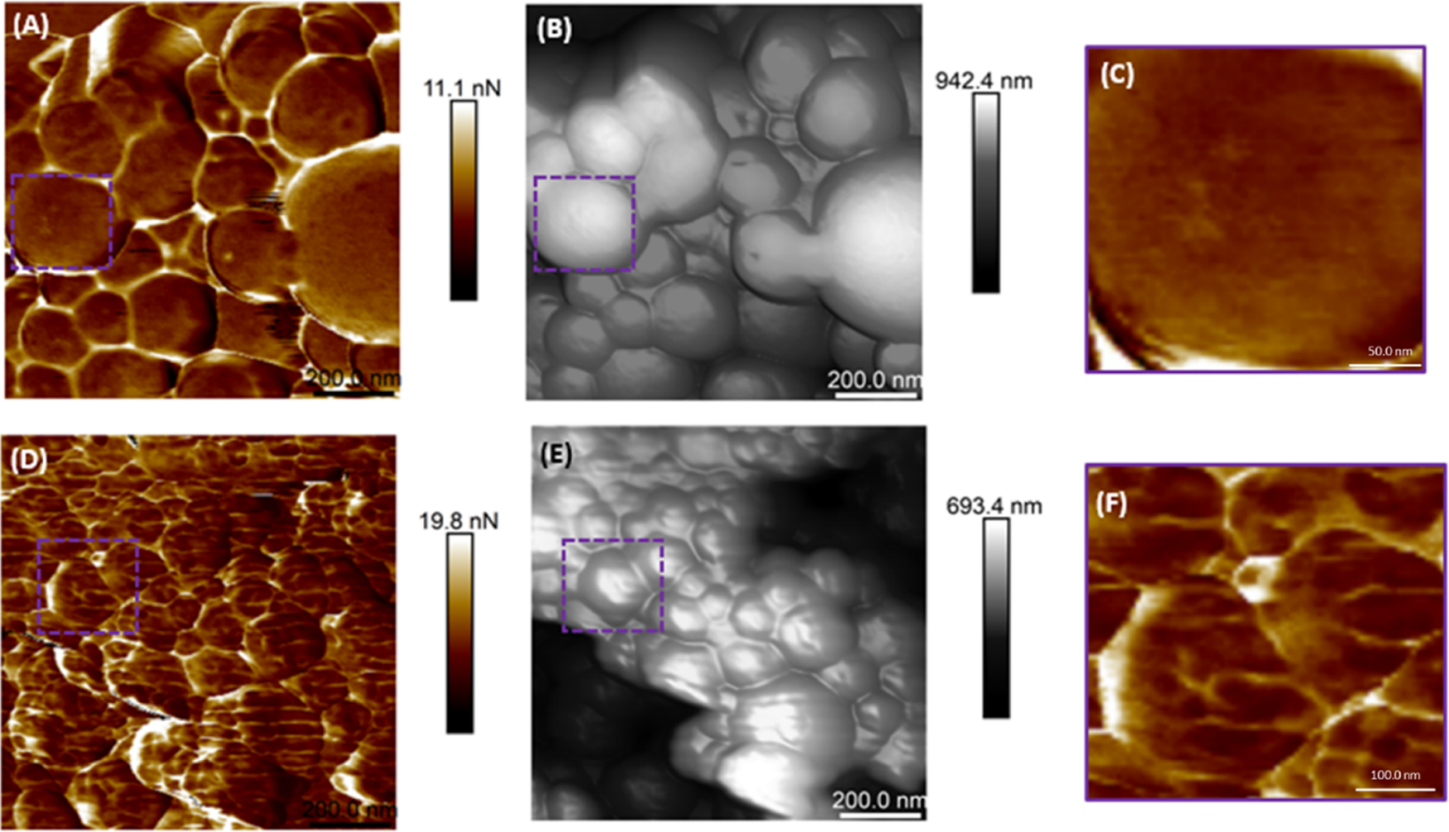
Adhesion force map (A) and corresponding height image of nonfunctionalized polymeric nanoparticles. Panel 6(C) corresponds to the scan region shown delimited by the squares positioned in panels (A) and (B). (C) Uniform adhesion forces distribution, corroborated by the box chart shown in panel (C) (right). (D, E) The adhesion map and height image, respectively, of the nanoparticles functionalized with atezolizumab antibody. Panel (F) corresponds to the details of the adhesion map evidenced by the squares positioned in panels (D) and (E). In this figure, it is possible to observe different patterns in the adhesion forces on the antibody-functionalized particles.

Figure 6 shows the adhesion force maps (Figure 6A,D) for each sample and the correlation with the height images (Figure 6B,E). It is possible to observe different adhesion patterns on particles functionalized with the atezolizumab antibody when compared to nonfunctionalized particles. The details of the adhesion maps are shown in Figure 6C,F. To confirm the maintenance of the pattern, scans at different angles were performed, confirming that the observed adhesion patterns were associated with changes in the particle surface due to atezolizumab antibody functionalization.

##### 3.2.2.3 Raman Spectroscopy

**Figure 7:**
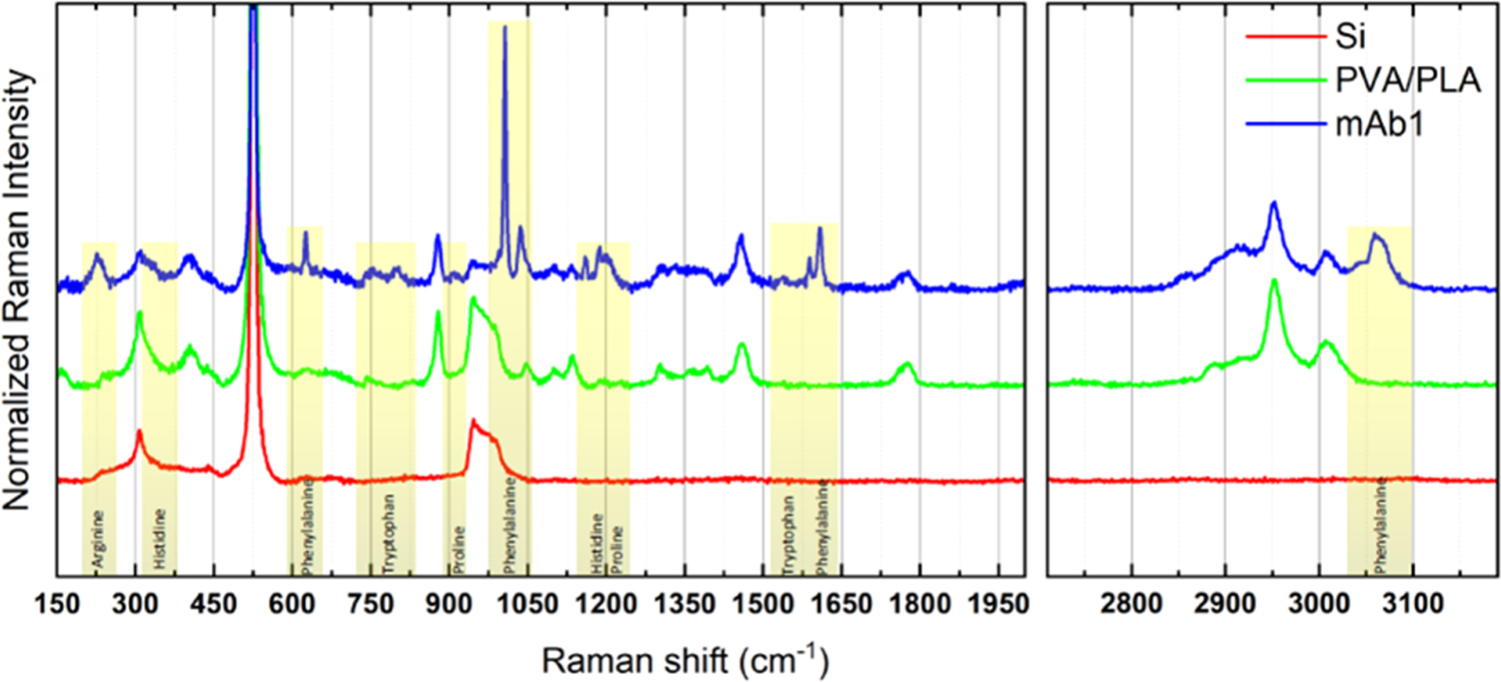
Raman spectra of empty polymeric nanoparticles (PVA/PLA, green), nanoparticles functionalized with the atezolizumab antibody (mAb1, blue), and only the silicon substrate on which both samples were deposited (red) for Raman measurement. The yellow stripes highlight the modes that appear in the nanoparticles after encapsulation of the mAb1. In the lower portion of each stripe, it is possible to observe each amino acid that constitutes the antibody molecular structure associated with each vibrational mode.

Figure 7 shows the Raman spectra of the silicon substrate (Si), the white sample (PLA/PVA), and nanoparticles with encapsulated atezolizumab antibody (mAb1).

The red spectrum was obtained on the Si substrate. It is possible to observe the contributions of the substrate in the white sample (PLA/PVA) and in the sample with the encapsulated antibody. The green spectrum was obtained on the white PLA/PVA nanoparticles. The observed modes are characteristic of these polymers. Separating the contributions of the substrate and the polymers of the nanoparticle shell, it is possible to observe changes in the spectrum from the different modes emphasized by the yellow stripes (blue spectrum). These modes are associated with the amino acids that constitute the atezolizumab antibody molecular structure, as detailed in each stripe. Some modes may be associated with more than one amino acid residue in the antibody structure since these amino acids (histidine, proline, phenylalanine, arginine, and tryptophan) have some vibrational modes awfully close to each other.^33^ This result corroborates with the changes observed by AFM in the nanoparticles, confirming the fact that the antibody is present in the nanoparticles.

#### 3.2.3 Preparation Yield and Encapsulation Efficiency of PLA/PVA/Atezolizumab Nanoparticles

The preparation yield and antibody binding efficiency of PLA/PVA/atezolizumab nanoparticles are given in Table 1.

There was no significant difference in the mean preparation yield of formulations with and without mAb (*p* < 0.05). In addition, different methods (M1–3) used within the scope of atezolizumab loading studies did not have a significant effect on the preparation efficiency (*p* < 0.05). However, different preparation methods used had a significant effect on encapsulation efficiency (%) (30.94–75.56%). The solvent evaporation technique, which has the highest binding efficiency value, was suitable in terms of particle size and surface properties and was suitable for the preparation of PLA/PVA/atezolizumab nanoparticle formulations.

### 3.3 Long-Term Stability Studies of Nanoparticles

Stability studies of formulations stored at 5 ± 3 °C (in the refrigerator) and 25 ± 5 °C, 60 ± 5% relative humidity and 40 ± 5 °C, 75 ± 5% relative humidity were carried out for 12 months at the initial stage and 1st, 3rd, 6th, and 12th months, and the results are given in Table 2.

**Table 2:** Stability Test Results (Particle Size as nm, PdI and ζ Potential as mV) of PLA/PVA/Atezolizumab Nanoparticle Formulations Prepared Using the Solvent Evaporation Method at the Initial Stage and 1st, 3rd, 6th, and 12th Months

	*T*_initial_	*T*_1month_	*T*_3month_	*T*_6month_	*T*_12month_
5 ± 3 °C	248.7 ± 2.116 nm	250.7 ± 0.990 nm	240.3 ± 2.916 nm	254.3 ± 2.023 nm	252.9 ± 1.058 nm
	0.132 ± 0.084	0.038 ± 0.050	0.179 ± 0.050	0.150 ± 0.048	0.097 ± 0.043
	–2.15 ± 0.27 mV	–1.65 ± ± 0.04 mV	–13.9 ± 1.98 mV	–5.1 ± 1.04 mV	–3.7 ± 1.98 mV
25 ± 5 °C	248.7 ± 2.116 nm	256.1±0.450 nm	282.4 ± 1.556 nm	290.6 ± 2.476 nm	299.4 ± 1.842 nm
	0.132 ± 0.084	0.045 ± 0.030	0.072 ± 0.076	0.104 ± 0.040	0.110 ± 0.078
60 ± 5%	–2.15 ± 0.27 mV	–0.31 ± 0.21 mV	–1.79 ± 0.38 mV	–3.75 ± 0.72 mV	–5.48 ± 1.76 mV
40 ± 5 °C	248.7 ± 2.116 nm	273.2 ± 1.875 nm	293.2 ± 0.141 nm	300.8 ± 2.548 nm	323.8 ± 3.261 nm
	0.132 ± 0.084	0.085 ± 0.053	0.078 ± 0.037	0.102 ± 0.048	0.151 ± 0.062
75 ± 5%	–2.15 ± 0.27 mV	–3.08 ± 1.04 mV	–1.23 ± 0.15 mV	–3.56 ± 0.09 mV	–5.32 ± 0.23 mV

According to Table 2, PLA/PVA/atezolizumab nanoparticular formulation was stable in all conditions and did not show any significant change in particle size, distribution, and ζ potential (*p* < 0.05).

### 3.4 Cell Culture Studies

#### 3.4.1 Cell Viability Studies

The number of microaggregate cells (non-blue-stained cells) and blue-stained cells was counted with the aid of a hemocytometer. Table 3 shows the number of all cells and the percentage of dead cells. The percentage of blue-stained cells was found to be below 15% for A549 and L929 cell suspensions. These values are available for seeding in later studies.^34^

**Table 3:** Summary of Cell Counting Results (Mean ± SD, *n* = 3)

	number of cells	
cell types	A549	L929
non-blue-stained cells	240.75 ± 2.25	260.50 ± 3.50
blue-stained cells	22.25 ± 1.50	25.25 ± 2.75
dead cells %	8.46 ± 0.50	8.84 ± 0.75

#### 3.4.2 Cytotoxicity–In Vitro Antitumoral Efficacy Study

**Figure 8:**
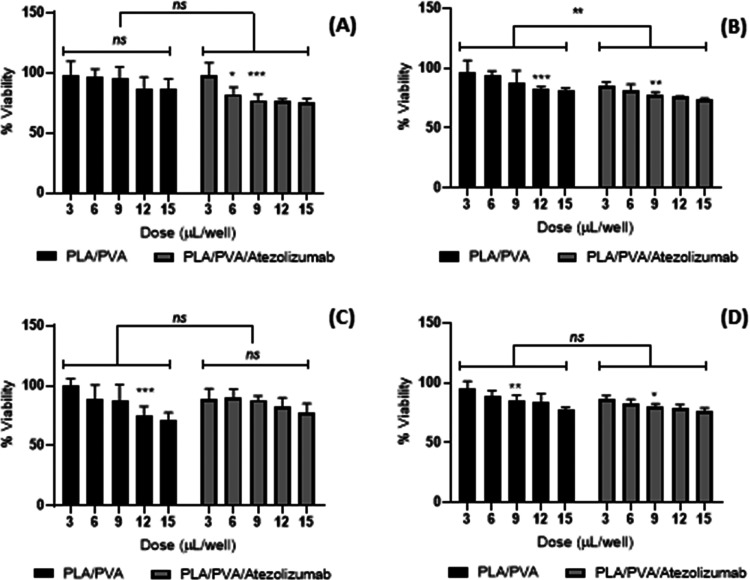
Alamar Blue assay of nanoparticles using five different activities (3–15 μL) (A) at 24 h in A549 cells, (B) at 48 h in A549 cells, (C) at 24 h in L929 cells, and (D) at 48 h in L929 cells. Data are expressed as mean ± standard deviation of three independent experiments, and *p < 0.05 and **p < 0.005 indicate a significant difference *vs* negative control.

The cytotoxic effects of PLA/PVA nanoparticles and PLA/PVA/atezolizumab nanoparticles at 24 and 48 h were investigated with A549 and L929 cells (Figure 8).

After a 24 h incubation period, the results of the cytotoxicity test with A549 cells were statistically examined (Figure 8A). According to the paired sample t-test analysis, there was a statistically significant difference between PLA/PVA nanoparticles and drug-loaded PLA/PVA/atezolizumab nanoparticles (*p* = 0.0168). Comparison between doses was performed using the two-way ANOVA analysis multiple comparison method (Figure 8A). In the evaluation, there was no statistically significant difference between increasing concentrations of PLA/PVA nanoparticles (*p* > 0.05). On the other hand, after 24 h of incubation, PLA/PVA/atezolizumab nanoparticles showed statistically significant toxicity at all doses (*p* = 0.001). The use of nanoparticles in lung cancer therapy has demonstrated good results. For instance, Shen et al.^35^ have used polyoxyethylene sorbitan oleate-modified hollow gold nanoparticles for lung therapy with expressive results. Also, Rosa et al.^36^ have developed polymeric dacarbazine microparticles radiolabeled with ^99m^Tc and ^223^Ra for lung cancer therapy and diagnosis with good results.

In the 48 h postincubation period, the results of the cytotoxicity test with A549 cells were statistically analyzed (Figure 8B). According to the paired sample t-test analysis, there was a statistically significant difference between PLA/PVA nanoparticles and drug-loaded PLA/PVA/atezolizumab nanoparticles (*p* = 0.0017). Application of PLA/PVA nanoparticles to A549 cells in increasing concentrations did not significantly affect cell viability at doses of 3 and 6 μL (*p* > 0.05) but showed a significant dose-dependent decrease in cell viability at doses of 9, 12, and 15 μL (*p* < 0.05). The dose response is an important parameter to elucidate toxicological aspects^37^ as drug dosage.^38^

Using L929 cells in an incubation time of 24 h, the results of the cytotoxicity test with L929 cells were statistically analyzed (Figure 8C). According to the paired sample t-test analysis, there was no statistically significant difference between PLA/PVA nanoparticles and drug-loaded PLA/PVA/atezolizumab nanoparticles (*p* > 0.05). Comparison between doses was performed using the two-way ANOVA analysis multiple comparison method (Figure 8C). In the evaluation, there was no statistically significant difference between increasing concentrations of drug-loaded PLA/PVA/atezolizumab nanoparticles (*p* > 0.05). The same has been observed in the 48 h postincubation period. The results of the cytotoxicity test with L929 cells were statistically analyzed (Figure 8D). According to the paired sample t-test analysis, there was a statistically significant difference between PLA/PVA nanoparticles and drug-loaded PLA/PVA/atezolizumab nanoparticles (*p* = 0.0119). Application of PLA/PVA nanoparticles to L929 cells in increasing concentrations did not cause an effect on cell viability at a dose of 3 μL, but at concentrations of 6 μL and higher, it was observed that it caused a dose-dependent decrease in cell viability. On application of PLA/PVA/atezolizumab nanoparticles to L929 cells in increasing concentrations, a dose-dependent decrease in cell viability was observed at all concentrations used.

Evaluation between PLA/PVA/atezolizumab and PLA/PVA nanoparticles groups was performed by the paired sample t-test. After an incubation period of 24 h, drug-loaded PLA/PVA/atezolizumab nanoparticles showed a statistically toxic effect on the cancer cell line A549 and significantly reduced cell proliferation (*p* = 0.0168). On the other hand, no statistically significant toxicity was observed between PLA/PVA/atezolizumab and PLA/PVA nanoparticles on healthy cell line L929 cells (*p* > 0.05). After an incubation period of 48 h, the difference between drug-loaded PLA/PVA/atezolizumab and drug-free PLA/PVA nanoparticles on A549 cells increased even more (*p* = 0.017). This suggests that it is more specific for the A549 cell line than the healthy cell line L929.

Although the drug-loaded PLA/PVA/atezolizumab nanoparticle formulation on the L929 cell line showed significant toxicity in the cytotoxicity study conducted for 48 h, it is predicted that this situation will not constitute significant toxicity considering the application time, plasma concentration, and removal time of the system developed *in vivo*.

The results in L929 cells demonstrated that the nanosystem is safe for healthy cells. This is corroborated by the findings of Helal-Neto et al.^39^ who have demonstrated that polymeric nanosystems are quite safe for biological application even at high concentrations. The safety of polymeric nanoparticles has also been demonstrated by Pinto et al.,^40^ where evaluating polymeric nanoparticles in pregnant rats showed no effect.

## 4 Conclusions

The results demonstrated that the developed PLA/PVA/atezolizumab nanoparticles had a spherical shape with a size of 230.6 ± 1.768 nm and a ζ potential of −2.23 ± 0.55 mV. Atezolizumab was entrapped in the nanoparticle with high encapsulation efficiency (80.58%), and the cytotoxic assay demonstrated the safety of the nanoparticle in L929 and the effect on A549. So, PLA/PVA/atezolizumab nanoparticles can be used as drug delivery systems and may represent an alternative for lung cancer diagnosis and therapy.
